# Association Between Oral Function and Oral-Related Quality of Life in Independent Community-Dwelling Elderly People in Taiwan

**DOI:** 10.3390/healthcare13111332

**Published:** 2025-06-03

**Authors:** Masayo Fukuda, Hiromi Izuno, Misao Sawada, Takako Ujihashi, Hinako Takano, Shoko Hori, Jumpei Okawa, Takahiro Ono, Kazuhiro Hori

**Affiliations:** 1 Division of Comprehensive Prosthodontics, Faculty of Dentistry, Graduate School of Medical and Dental Sciences, Niigata University, Niigata 951-8514, Japan; m-fukuda@kobe-tokiwa.ac.jp (M.F.); tkn175haru121@dent.niigata-u.ac.jp (H.T.); horisho@dent.niigata-u.ac.jp (S.H.); jookawa@dent.niigata-u.ac.jp (J.O.); 2 Department of Oral Health Science, Faculty of Health Sciences, Kobe Tokiwa University, Kobe 653-0838, Japan; m-sawada@kobe-tokiwa.ac.jp (M.S.); t-ujihashi@kobe-tokiwa.ac.jp (T.U.); 3 Department of Oral Health Sciences, Otemae College, Nishinomiya 662-8552, Japan; h-izuno@otemae.ac.jp; 4 Department of Oral Health Sciences, Faculty of Nursing and Health Care, BAIKA Women’s University, Ibaraki 567-8578, Japan; 5 Department of Geriatric Dentistry, Osaka Dental University, Osaka 540-0008, Japan; ono-t@cc.osaka-dent.ac.jp

**Keywords:** oral-related quality of life, oral function, independent elderly person, tongue

## Abstract

Background/Objectives: Oral health is an important element of overall health and quality of life. However, few studies have evaluated the association between quality of life (QOL) and oral function, including tongue and lip movements. This study aimed to investigate the relationship between oral-related QOL and oral function, including tongue and lip movements, in independent elderly people. Methods: The participants were 143 community-dwelling elderly people in Taiwan (40 men, 103 women; 76.4 ± 6.4 years). We used the General Oral Health Assessment Index (GOHAI) to evaluate oral-related QOL. The items used to evaluate oral function were as follows: the speed of tongue movement from side to side, maximum tongue pressure, maximum lip pressure, the repeated saliva swallowing test, oral diadochokinesis (speed and dexterity when pronouncing /pa/, /ta/, and /ka/), and masticatory performance. In addition, we surveyed the number of remaining teeth, denture use, and awareness of problems at mealtimes. For the analysis, we compared the GOHAI score for each group, divided by cutoff values. Results: The univariate analysis revealed significant differences in the GOHAI score according to denture use, the number of remaining teeth, the state of occlusal support areas, the speed of tongue movement from side to side, oral diadochokinesis /ta/ and /ka/, and masticatory performance. The logistic regression analysis revealed that oral diadochokinesis /ka/ was a significant explanatory variable for low GOHAI scores (odds ratio = 13.145). Conclusions: Our results imply that lower oral-related QOL was associated with slow motor function at the rear area of the tongue.

## 1. Introduction

In aging societies, health initiatives aim to reduce the difference between the average life expectancy and healthy life expectancy and to maintain a healthy state throughout life through initiatives such as disease prevention, health promotion, and nursing care for the elderly. Thus, the promotion of health and enhancement of quality of life are important not only for medical treatment but also for society and the economy.

There is growing interest in using quality of life (QOL) as an index of the outcome of such measures for elderly people. The concept of QOL is widely recognized in the fields of medicine, healthcare, and welfare, and the maintenance and improvement of QOL are seen as particularly important goals with respect to elderly populations. QOL evaluations include health-related QOL, which is mainly carried out in the medical field and aims to measure health by objective or subjective scales, and subjective QOL, which can evaluate individuals in terms of their physical, psychological, social, and environmental status [1,2]. In the 1990s, oral-related QOL was proposed for use in the field of oral health. Oral-related QOL comprises functional, psychological, social, and pain/discomfort components, and the main indices are the General Oral Health Assessment Index (GOHAI), the Dental Impact Profile, the Oral Health Impact Profile, and the Subjective Oral Health Status Indicator. The validity and reproducibility of these indices have been evaluated [3,4]. Prior studies in the field of oral health have reported that QOL is related to decreased feeding and swallowing function, the number of remaining teeth, denture status, the living environment, and poor nutrition in elderly people [5,6].

The mobility and motor dexterity of the perioral organs play an important role in feeding and swallowing, mastication, and articulation, and maintaining such oral function is considered to be related to the maintenance or improvement of QOL. Oral function includes factors important to mastication and swallowing such as the function of the tongue and perioral muscles. Previously, we studied the relationship between physical fitness and oral function among Japanese independent elderly participants, with the goal of providing support for the prevention of long-term medical care. As a result of that study, we reported that the evaluation of tongue and lip movements might augment assessments of physical function in elderly people [7]. Taiwan, along with Japan, is facing the one of the fastest aging rates in the world. However, to the best of our knowledge, no studies have examined the relationship between oral-related QOL and oral function via objective measurements of tongue and lip function in elderly people.

We hypothesized that oral function, including that of the tongue and lips, is related to the oral-related QOL in elderly people. In a rapidly aging society, such as Japan or Taiwan, surveys of the relationship between oral function and QOL are likely to be effective in clarifying the importance of oral function to overall health. Therefore, in the present study, we investigated the relationship between evaluations of oral function and the GOHAI among independent elderly persons living in the community in Taiwan. We sought to clarify the relationship between oral-related QOL and the number of remaining teeth, masticatory performance, and oral function including tongue and lip movements.

## 2. Materials and Methods

### 2.1. Participants

The participants included 143 independent elderly persons (40 men, 103 women; mean age 76.4 ± 6.4 years, range 65–91 years) participating in long-term care prevention classes at the Taichung YMCA and Taipei YMCA. The data were collected from August 2016 to April 2017. The inclusion criteria were as follows: all participants used a local facility for the support of older people and were recruited via the Taipei YMCA and Taichung YMCA. In addition, all of the participants were independent in daily life, were over the age of 65, and were available to participate on the survey day. The exclusion criteria were a history of cerebrovascular disease, neuromuscular disease, dementia, or arthritis.

Prior to engaging in this study, the participants were given a full explanation regarding the objectives and methods of the research in document form and verbally in Chinese. They all provided written consent to participate in this study. This study was carried out with the approval of the Ethics Committee of Baika Women’s University (approval no. 0010-0091).

### 2.2. Items for Evaluation of Oral Function

Oral function was evaluated using the following six items, which were used in a prior study by Izuno et al. [7]. In the case of denture users, measurements were taken with the dentures fitted (Table 1). The measurements were taken at least one hour after the meal.

#### 2.2.1. Speed of Tongue Movement from Side to Side

Participants were instructed to move the tip of their tongue as fast as possible from side to side between the corners of the mouth. The time (sec) required to complete 10 movements from one side and back was measured using a stopwatch [7].

#### 2.2.2. Maximum Tongue Pressure

The maximum pressure exerted when pressing with the tongue (maximum tongue pressure) was measured using a tongue pressure measurement device with a balloon probe (JM-TPM, JMS Co., Ltd., Hiroshima, Japan) [8,9]. While sitting in a relaxed position, participants were instructed to position the balloon on the probe at the front of their palate and then to press the balloon against their palate with their tongue for 7 s, during which time the maximum pressure was recorded. Measurements were taken 3 times, each with a 1 min rest interval, and the mean value (kPa) was taken as the representative value.

#### 2.2.3. Maximum Lip Pressure

Maximum lip pressure was measured using a tongue pressure measurement device with a balloon probe (JM-TPM) [10]. The participant was instructed to place the balloon probe between their upper and lower lips and then to compress the balloon with their lips for 7 s, during which time the maximum pressure was recorded. Measurements were taken 3 times, each with a 1 min rest interval, and the mean value (kPa) was taken as the representative value.

#### 2.2.4. Oral Diadochokinesis

The participants were instructed to say the syllable /pa/ as quickly and clearly as possible for 5 s [11,12], which was counted using a Kenkokun Handy digital counter (T.K.K.3350, Takei Scientific Instruments Co., Ltd., Niigata, Japan). The syllables /ta/ and /ka/ were measured in the same manner, and the number of utterances per second was taken as the representative value.

#### 2.2.5. Masticatory Performance

We used gummy jellies to measure masticatory performance (UHA Mikakuto Co., Ltd., Osaka, Japan). We asked the participants to chew the gummy jellies, and we evaluated the degree of pulverization after 30 rounds of chewing using the visual scoring method [13].

### 2.3. Index of Oral-Related QOL

We used the Chinese version of the GOHAI as an index of oral-related QOL [14,15,16]. The minimum score was 12 and the maximum score was 60, with a higher score indicating higher oral-related QOL.

### 2.4. Other Evaluation Items

The number of remaining teeth and denture use were confirmed by a dentist. Eichner’s classification was determined based on the state of remaining occlusal support areas [17,18].

Furthermore, we used the repetitive saliva-swallowing test (RSST) to screen for dysphagia. While sitting in a relaxed position, the participant was instructed to repeatedly swallow saliva as many times as possible for 30 s, and the number of swallows was measured [12,19,20].

Prior to the measurements of oral function, the height and weight of each participant was measured and participants completed a self-administered questionnaire in Chinese that asked about the history of any present illness, current medications, exercise habits, choking during meals, sputum in the throat during or after meals, and dry mouth.

### 2.5. Analysis Methods

First, we used a univariate analysis to examine the participant characteristics and the relationships between GOHAI scores and each of the surveyed factors. A cutoff value was set for each factor (Table 1). The participants were then divided into two groups according to their cutoff value, and GOHAI scores were compared between groups. The cutoff value for age was 75 years, which is the age at which individuals are considered to be latter-stage elderly. Based on the diagnostic standards for “oral hypofunction” defined by the Japanese Society of Gerodontology [21], the cutoff value for the number of remaining teeth was 20, that for tongue pressure was 30 kPa, and that for oral diadochokinesis was 6 times/sec. The cutoff value for RSST was 3 times/sec, which is the screening value for dysphagia [19,20]. The cutoff value for masticatory performance was set at a score of 3 on the basis of the diagnostic standards for “oral hypofunction” defined by the Japanese Society of Gerodontology [21]. As there are no standard values for the speed of tongue movement from side to side or for lip pressure, the median values were taken as cutoff points (Table 1). As none of the data conformed to a normal distribution, analyses were carried out using the Mann–Whitney U test and the Kruskal–Wallis test. We then conducted a multivariate analysis to search for factors related to decreased oral-related QOL. We performed a logistic regression analysis with the participants divided into two groups on the basis of the GOHAI score. Specifically, GOHAI scores within the first quartile were set as the response variable and items with significant differences in the univariate analysis were set as explanatory variables. Statistical processing was carried out using SPSS ver. 23 for Windows (IBM Japan, Tokyo, Japan), with a 5% significance level.

## 3. Results

### 3.1. Participant Characteristics

The participant characteristics are shown in Table 2. The mean age of the participants was 76.4 ± 6.4 years. The questionnaire results indicated that out of the 143 participants, 12 (8.4%) reported choking during meals, 14 (9.8%) reported sputum in the throat during meals, 19 (13.3%) reported mouth dryness during meals, 112 (78.3%) reported some kind of disease, 112 (78.3%) reported taking medicine, and 129 (90.2%) reported exercise habits. The mean number of remaining teeth was 18.2 ± 9.2 (median 22 teeth), and 61 participants (42.7%) used dentures.

The mean age for men was 76.4 ± 5.7 years and that for women was 76.4 ± 6.3 years, with men having a significantly greater height and body weight compared with women. Although there were more women than men in this cohort, other than height and body weight, there were no significant gender-based differences for any items.

### 3.2. Relationship Between Each Factor and GOHAI Score

The median GOHAI score for all of the participants was 49.0. Table 3 shows a comparison between the GOHAI scores for the two groups for each factor. Significant differences were found for denture use, the number of remaining teeth, the Eichner classification, the speed of tongue movement from side to side, oral diadochokinesis /ta/, oral diadochokinesis /ka/, and masticatory performance (*p* < 0.05 for all). No significant differences in GOHAI score were found for choking during meals, subjective feelings of mouth dryness, sputum in the throat during or after meals, tongue pressure, lip pressure, RSST, or oral diadochokinesis /pa/.

Table 4 shows the results of the logistic regression analysis. Analysis was carried out using the speed of tongue movement from side to side, oral diadochokinesis /ta/, oral diadochokinesis /ka/, masticatory performance, the Eichner classification, the number of remaining teeth, and denture use, which showed significant differences in the univariate analysis, as explanatory variables. Oral diadochokinesis /ka/(odds ratio = 13.145, 95% confidence interval 1.719–100.51, *p* = 0.013) was selected as a variable affecting oral-related QOL (in the model χ^2^ test: *p* < 0.01; percentage of correct classification, 73.0%).

## 4. Discussion

In a super-aging society, initiatives are needed that target disease prevention, health promotion, and the prevention of long-term care for elderly people. Accordingly, measures that promote health and improve QOL are essential. In the present study, we investigated the relationship between oral-related QOL and oral function, including tongue and lip movements, in independent elderly persons actively participating in long-term care prevention activities.

The mean age of the participants in the present study was 76.4 ± 6.4 years, which is higher than the mean age of participants in previous studies of independent elderly persons in Japan [22,23,24,25], including that in the study by Izuno et al. [7]. Almost all of the participants exercised 1–2 times or more per week. Thus, while the present participants were older than those in previous studies, they were actively engaged in activities to prevent the need for care.

The GOHAI is one of the most frequently used tools to assess oral health-related quality of life [26]. The GOHAI score results in the present study were lower than standard values [27] for the Japanese population (70–79 years: mean 50.8 ± 8.8, median 52.8). Although standard GOHAI scores for Taiwan are not available, prior studies in Taiwan reported scores of 44.3 ± 8.8 [15] and 48.3 ± 0.4 [28] for community-dwelling elderly adults (mean age 76.0 years), and 47.8 ± 0.4 [14] for removable denture wearers (mean age 75.07 years), which is consistent with the results of the present study.

Our previous analysis indicated that the participant group in the present study included many latter-stage elderly persons who were actively engaged in long-term care prevention activities, maintained relatively good oral health, and had lower GOHAI scores than Japanese people but had scores that were comparable to those of the standard population in Taiwan.

Previous studies have reported a significant relationship between oral-related QOL and the number of remaining teeth [5,29,30]. In the present study, we found a significant difference in GOHAI scores between the group with more remaining teeth (≥20) and the group with fewer remaining teeth (≤19). Furthermore, the group with more occlusal-supporting areas according to the Eichner classification had significantly higher GOHAI scores than the group with fewer occlusal-supporting areas. The results regarding denture use showed that denture users had significantly lower GOHAI scores than non-denture users, which is consistent with other studies [5]. Almost all of the present participants who were considered to be in need of dentures did in fact use dentures, although we did not carry out a qualitative evaluation of dentures. From these results, we could not identify which the lower GOHAI score was due to excessive tooth loss or low-quality dentures. Therefore, a future investigation is needed to examine the oral-related QOL of denture users.

As with prior studies, GOHAI scores significantly varied according to masticatory performance. Many studies have reported a relationship between oral-related QOL and subjectively measured masticatory performance, i.e., that evaluated by means of a questionnaire [31,32]. This decrease in oral-related QOL was probably due to discomfort and dissatisfaction at mealtimes induced by difficulty with mastication, as well as the resulting adverse effect on nutritional intake. As we used an objective measure of masticatory performance in the present study, i.e., chewing gummy jellies, we are confident that the resulting significant difference in GOHAI scores between groups indicates the presence of a relationship between oral-related QOL and masticatory performance.

Furthermore, we found that GOHAI scores significantly differed according to the speed of tongue movement from side to side, oral diadochokinesis /ta/, and oral diadochokinesis /ka/. Oral diadochokinesis /ta/ is used to evaluate the motor function of the front of the tongue, and oral diadochokinesis /ka/ is used to evaluate the motor function of the rear and base of the tongue. Thus, our findings suggest that skilled movement of the tongue, including the speed of tongue movement from side to side, is related to oral-related QOL. The tongue is involved in mastication, pronunciation, and swallowing, as well as the formation and transport of the food bolus, and it also contributes to the retention and stability of dentures. Given these extremely important roles, we were not surprised to find that tongue function was intimately linked to oral-related QOL in elderly people.

Although prior studies have reported reduced oral-related QOL in persons who are conscious of dryness of the mouth [29,33], we found no significant differences in the present study. Moreover, we found no significant QOL-related differences in the rates of subjective symptoms of choking during meals or sputum in the throat during meals. This is probably because the present participants were relatively healthy elderly people who actively participated in long-term care prevention activities, and these subjective symptoms were relatively rare in the study population. In addition, we found no significant differences in the GOHAI score according to tongue pressure, lip pressure, RSST, or oral diadochokinesis /pa/, although many participants had scores that were lower than the standard values for “oral hypofunction” [21]. This indicates that even among independent elderly persons who actively participate in long-term care prevention activities, many individuals have deteriorated oral function, which is directly associated with age and the need for care [21,34], and may impact other functions [9].

A logistic regression analysis of factors relating to deterioration of oral-related QOL revealed a relationship between oral-related QOL and oral diadochokinesis /ka/. Oral diadochokinesis is an examination method that comprehensively evaluates the speed of and ability for tongue and lip movements, and /ka/, in particular, reflects the motor function of the rear and root of the tongue. Tongue motor function greatly influences food ingestion and swallowing. The previous studies also indicated that diadochokinesis /ka/ was related to the swallowing function [35,36]. After food has been introduced into the mouth, the movements of the tip of the tongue and the dorsal region of the tongue are involved in forming the bolus during mastication. Next, when the bolus is transported to the pharynx, the posterior tongue comes into contact with the soft palate and the root of the tongue moves downward and forward. This opens the hypopharyngeal region so that the bolus can pass, inducing swallowing.

The tongue is also involved in articulation, and different sounds are made through precise movements of the tip, forward part, central part, and root of the tongue, using articulation points such as the palate, gums, and teeth. Thus, deterioration of the motor function of the posterior region or root of the tongue can lead to impaired mastication, swallowing, and articulation. For elderly people, eating tasty, enjoyable, and safe meals for their entire lives is important to maintaining a high level of life function. The finding that oral diadochokinesis /ka/ was a factor associated with oral-related QOL in the present study may be related to deterioration in the function of the rear part or root of the tongue, lessening the pleasure derived from eating and speaking to others. However, it is unclear whether this result directly indicates that the other items not selected are not related to oral-related QOL. It should be necessary to continue the survey with a larger number of participants.

Our results show that even among elderly independent people who actively participate in long-term care prevention activities, many individuals had deteriorating oral function. This finding highlights the importance of early detection and early responses to deteriorations of oral function for the maintenance and improvement of QOL in elderly people.

As our survey was cross-sectional, it was not possible to identify causal relationships among the variables. In addition, we did not consider the impact of living environment, such as whether the participants lived in urban areas or lightly populated areas. As the present study was carried out in Taiwan, the local characteristics of the region should be taken into account. We were not able to make a direct comparison between individuals in Taiwan and those in Japan due to differences in the age of the elderly participant groups. To further clarify the relationship between QOL and oral function in elderly persons, continuous surveys in both Japan and Taiwan are necessary.

## 5. Conclusions

To survey the association between QOL and oral function in elderly people, we examined the relationship between oral-related QOL and oral function. This study suggested an association between oral-related QOL and tongue motor function. This result implies that oral function was related to the oral-related QOL in elderly people.

## Figures and Tables

**Table 1:** Investigated variables and cutoff values.

Items	Cutoff Value
Age	≥75 y/65~74 y
Number of remain teeth	≥20/≤19
Tongue movement from side to side (sec)	≥8.5/<8.5
Tongue pressure (kPa)	≥30/<30
Lip pressure (kPa)	≥2.6/<2.6
RSST (/30 s)	≥3/≤2
Oral diadochokinesis /pa/ /ta/ /ka/ (/sec)	≥6/≤5
Masticatory efficiency	≥3/≤2

**Table 2:** General and dental status of surveyed participants by gender.

		All (*n* = 143)		Male (*n* = 40)		Female (*n* = 103)		*p*-Value	
Age	Mean (SD), years	76.4	(6.4)	76.4	(5.7)	76.4	(6.3)	0.99	a
Height	Mean (SD), cm	156.4	(8.3)	165.5	(6.3)	153.1	(6.1)	<0.01	**a
Weight	Mean (SD), kg	59.7	(11.1)	66.4	(10.6)	57.2	(10.2)	<0.01	**a
BMI	Mean (SD), kg/m^2^	24.4	(4.1)	24.2	(3.2)	24.5	(4.4)	0.67	a
Number of remaining teeth	Mean (SD)	18.2	(9.2)	17.6	(8.4)	18.5	(9.5)	0.65	a
GOHAI score	Median (QD)	49.0	(4.0)	48.0	(5.5)	49.0	(4.0)	0.84	a
Denture wearer	*n* (%)	61	(42.7)	21	(52.5)	40	(38.8)	0.14	b
Choking during meal	*n* (%)	12	(8.4)	3	(7.5)	9	(8.7)	1.00	c
Sputum at meal	*n* (%)	14	(9.8)	4	(10.0)	10	(9.7)	1.00	c
Mouth dryness at meal	*n* (%)	19	(13.3)	5	(12.5)	14	(13.6)	0.86	b
Disease being treated	*n* (%)	112	(78.3)	32	(80.0)	80	(77.7)	0.76	b
Regular medication	*n* (%)	112	(78.3)	31	(77.5)	81	(78.6)	0.88	b
Regular exercise	*n* (%)	129	(90.2)	36	(90.0)	93	(90.3)	1.00	c
Tooth brushing more than three times a day	*n* (%)	71	(49.7)	15	(37.5)	56	(54.3)	0.02	b
Current smoking	*n* (%)	8	(5.6)	5	(12.5)	3	(2.9)	0.04	c

** *p* < 0.01, a: by Mann–Whitney U test, b: by Chi-square test, c: by Fisher’s exact test. SD; standard deviation, QD; quartile deviation.

**Table 3:** Relationship between each factor and GOHAI scores.

Factor	Item	*n*	Median GOHAI Score	QD	*p*-Value	
Age	65–74 y	58	48.0	4.5	0.58	a
	75+ y	85	49.0	4.5		
Sex	Male	40	48.0	5.0	0.84	a
	Female	103	49.0	4.0		
Denture wearer	yes	61	48.0	5.0	<0.01	**a
	no	82	51.0	4.5		
Choking during meal	yes	12	47.5	5.0	0.27	a
	no	131	49.0	4.0		
Sputum at meal	yes	14	48.0	4.5	0.71	a
	no	129	49.0	4.0		
Mouth dryness at meal	yes	19	48.0	3.0	0.70	a
	no	124	49.0	4.0		
Number of remaining teeth	≤19	63	48.0	5.0	0.04	*a
	≥20	78	50.0	4.5		
State of occlusal supporting area	A	56	51.0	5.0	0.01	*b
(Eichner’s classification)	B	54	47.0	5.5		
	C	33	48.0	3.0		
Tongue movement from side to side (sec)	<8.5	70	50.5	4.5	0.01	*a
	≥8.5	73	48.0	5.0		
Tongue pressure (kPa)	<30	80	48.0	4.0	0.90	a
	≥30	63	49.0	3.5		
Lip pressure (kPa)	<2.6	69	48.0	5.5	0.06	a
	≥2.6	74	49.5	3.5		
RSST (/30s)	≤2	24	48.5	4.0	0.50	a
	≥3	119	49.0	4.0		
Oral diadochokinesis /pa/ (/sec)	≤5	104	48.0	4.0	0.21	a
	≥6	39	51.0	5.0		
Oral diadochokinesis /ta/ (/sec)	≤5	102	48.0	4.0	0.03	*a
	≥6	41	52.0	5.5		
Oral diadochokinesis /ka/ (/sec)	≤5	114	48.0	4.5	<0.01	**a
	≥6	29	52.0	3.5		
masticatory performance	≤2	38	47.0	4.0	0.03	*a
	≥3	105	50.0	4.5		

a: * *p* < 0.05, ** *p* < 0.01 by Mann–Whitney U test. b: * *p* < 0.05 by Kruskal–Wallis test. RSST: repeated saliva swallowing test, QD; quartile deviation.

**Table 4:** Multivariate logistic regression models for GOHAI scores.

Objective Variable	Explanatory Variable	B	*p*	Odds Radio	95% [CI]
GOHAI score 12–44/45–60	oral diadochokinesis /ka/	2.576	0.013	13.145	1.719–100.510
	constant	−1.856	0.090	0.156	

Model χ^2^ test: *p* < 0.01l; percentage of correct classification: 73.0%. Adjusted variables: number of remaining teeth, denture use, Eichner classification, masticatory efficiency, tongue movement from side to side, oral diadochokinesis /ta/, and oral diadochokinesis /ka/.

## Data Availability

The data presented in this study are available on request from the corresponding author. The data are not publicly available due to ethical restrictions.
